# Risk of breast, ovary, and uterine corpus cancers among 85 268 women with AIDS

**DOI:** 10.1038/sj.bjc.6603282

**Published:** 2006-07-25

**Authors:** J J Goedert, C Schairer, T S McNeel, N A Hessol, C S Rabkin, E A Engels

**Affiliations:** 1 Viral Epidemiology Branch, Division of Cancer Epidemiology and Genetics, National Cancer Institute, Rockville, MD 20892, USA; 2 Biostatistics Branch, Division of Cancer Epidemiology and Genetics, National Cancer Institute, Rockville, MD 20892, USA; 3 Information Management Services, Inc., Silver Spring, MD 20904, USA; 4 Department of Medicine, University of California, San Francisco, CA 94122, USA

**Keywords:** breast cancer, endometrial cancer, ovarian cancer, human immunodeficiency virus (HIV), acquired immunodeficiency syndrome (AIDS), risk factors

## Abstract

By linking HIV/AIDS and cancer surveillance data in 12 US regions, breast and reproductive cancer risks with AIDS were compared to those in the general population. Trends in standardized incidence ratios (SIRs) were assessed by CD4 count, AIDS-relative time, and calendar time. Standardized incidence ratios were indirectly adjusted for cancer risk factors using data from AIDS cohort participants and the general population. With AIDS, 313 women developed breast cancer (SIR 0.69, 95% confidence interval (CI) 0.62–0.77), 42 developed ovary cancer (SIR 1.05, 95% CI, 0.75–1.42), and 31 developed uterine corpus cancer (SIR 0.57, 95% CI, 0.39–0.81). Uterine cancer risk was reduced significantly after age 50 (SIR 0.33). Breast cancer risk was reduced significantly both before (SIR 0.71) and after (SIR 0.66) age 50, and was lower for local or regional (SIR 0.54) than distant (SIR 0.89) disease. Breast cancer risk varied little by CD4 count (*P*_trend_=0.47) or AIDS-relative time (*P*_trend_=0.14) or after adjustment for established cancer risk factors. However, it increased significantly between 1980 and 2002 (*P*_trend_=0.003), approaching the risk of the general population. We conclude that the cancer deficit reflected direct or indirect effects of HIV/AIDS and that anti-HIV therapy reduced these effects.

Increasing numbers of women have been infected with human immunodeficiency virus (HIV) and now are living with the acquired immunodeficiency syndrome (AIDS). The effects of AIDS on women, particularly in the era of highly active antiretroviral therapy (HAART), are not well defined. In the era before HAART, the risks of developing Kaposi sarcoma, anal cancer, and perhaps lymphoma were lower for women than for men with AIDS (Beral and Newton, 1998; Goedert, 2000). Other than cervical cancer, no differences by gender have been reported for the few other malignancies found in excess among people with AIDS (PWA) (Goedert *et al*, 1998; Frisch *et al*, 2001).

Breast cancer may occur less often in women with AIDS than in the general population (Goedert *et al*, 1998; Frisch *et al*, 2001). Ovary and uterine corpus cancers have not been associated with AIDS or other immune deficiencies, but weak associations or susceptible subgroups may have been overlooked because these malignancies are relatively rare. No previous study has evaluated the effects of improving anti-HIV therapies or whether differences in cancer incidence might merely reflect differences in known risk factors for cancer.

Using population-based data, we have investigated whether the risk of breast, ovary, or uterine corpus cancers differ for women with AIDS, whether breast cancer risk differs with increasing severity or duration of immune deficiency, or with increasing availability and efficacy of anti-HIV therapies; also whether menopause or selected risk factors modify or explain any associations.

## MATERIALS AND METHODS

### Detection and definition of cancers

From 2003 through 2005, we linked HIV/AIDS and cancer registration data (including name, race, sex, dates of birth and death, and, where available, social security numbers) in 12 regions of the US to identify cancers arising among PWA (Goedert *et al*, 1998; Frisch *et al*, 2001; Borges *et al*, 2001). Ethical and legal reviews at all participating HIV/AIDS and cancer registries ensured that patient confidentiality was maintained.

This study describes the cancer profile of women diagnosed with AIDS from ages 15 to 91 years, referred to below as the registry population. Time at risk, expressed as person-years, was calculated from the start to the end of complete cancer registration in the particular area, no earlier than 60 months before nor later than 120 months after AIDS onset, and censored at death if less than 120 months after AIDS. Although migration could not be determined, both the HIV/AIDS and cancer registries used the National Death Index to ascertain deaths, including those occurring outside the registration areas. The current study included only invasive cancers, coded according to the International Classification of Diseases for Oncology (ICD-O), and analysed by site using the Surveillance, Epidemiology and End Results program's ‘Site recode with KS and mesothelioma’ (Fritz *et al*, 2000; Ries *et al*, 2005). Individual cancer sites (ICD-O C50, C54, and C56.9) were analysed after excluding tumours at those sites that had unspecified, KS or NHL histology (codes 8000–8005, 9140, and 9050–9055 or 9590–9989, respectively).

### Measures of relative risk

Detailed statistical methods are provided in the appendix. Briefly, we used previously described methods to calculate the standardized incidence ratio (SIR), which is the ratio of observed to expected cancers derived from contemporaneous, racial/ethnic ancestry-, age-, and registry-specific population-based incidence rates (Goedert *et al*, 1998; Frisch *et al*, 2001). For presentation, ancestry was grouped as African and other. Expected numbers of cancer were calculated as the sum of stratum-specific products of background cancer incidence and person-months at risk among the PWA and were discounted for periods before AIDS onset to account for reduced survival following a cancer diagnosis (Breslow and Day, 1987; Grulich *et al*, 1999; Frisch *et al*, 2000; Engels *et al*, 2003). We calculated 95% confidence intervals (CI) assuming a Poisson distribution of the observed cancers (Breslow and Day, 1987). For an overall SIR estimate, we used the 15-year period from 60 months before to 120 months after AIDS onset. All subgroup and trend analyses were preplanned. To estimate cancer risk before and after menopause, SIRs were calculated by age at cancer diagnosis, grouped as <50 and ⩾50 years.

We used Poisson modeling and a two-sided score test (Goedert *et al*, 1998; Frisch *et al*, 2001) to evaluate change in SIR across six AIDS-relative time intervals, as presented. We excluded the AIDS onset period (6 months before to 3 months after AIDS onset) from the trend test to reduce the influence of ascertainment bias from the generally increased diagnostic activity around the time of AIDS diagnosis. A similar approach was used to evaluate change in SIR across CD4 lymphocyte count at AIDS onset (limited to years 1990–2002 because CD4 data generally were not available before 1990) and across calendar intervals (limited to 4–27 months after AIDS onset to focus on an interval when follow-up would be most complete). In sensitivity analyses (not presented), we found no difference in the trend in breast cancer SIR with an alternative time interval (4–60 months after AIDS onset) or calendar intervals (1980–1986, 1987–1995, 1996–2002). To further assess trends in SIR, we used Poisson regression to fit linear models for breast cancer risk across calendar time (1980–2002). Although women with HIV/AIDS had lower fertility and BMI in the pre-HAART era, (Lee *et al*, 2000; Forsyth *et al*, 2002), the breast cancer deficit with AIDS was minimally altered by adjustment for these factors.

### Indirect adjustment

We used indirect methods to adjust for the different prevalence of selected cancer risk factors in PWA as compared to the general population (Steenland and Greenland, 2004). Details are provided in the Appendix. For the general US population, prevalence data on age at first live birth, parity, body mass index (BMI), cigarette smoking, and oral contraceptive use were derived from a weighted average of women in the 1987 (*n*=24 898) and 2000 (*n*=24 503) National Health Interview Surveys (NHIS) (Centers for Disease Control and Prevention, 2002). For women with AIDS, prevalence data on these risk factors were obtained from female participants with AIDS (henceforth referred to as AIDS patients) in the AIDS Cancer Cohort Study (*n*=483) or the Women's Interagency HIV Study (WIHS, *n*=693) (Barkan *et al*, 1998; Nawar *et al*, 2005). These two studies included subjects from six of the HIV/AIDS registry areas (New York City, Los Angeles, San Francisco, Florida, Massachusetts, and Connecticut), and the demographic characteristics of the AIDS patients resembled those of our registry population and of the national female HIV/AIDS population (Bacon *et al*, 2005). The majority (59%) of participants in WIHS were below the Federal poverty level, and 13% did not reside in a house or apartment (Barkan *et al*, 1998). We excluded AIDS patients and NHIS women who had a history of breast cancer.

For breast cancer, we adjusted for the effects of age at first live birth or nulliparity and BMI with age-specific relative risks (RRs) (MacMahon *et al*, 1970; Ballard-Barbash and Swanson, 1996; Friedenreich, 2001; Althuis *et al*, 2003). In a sensitivity analysis that yielded similar results (not presented), we used African American-specific RRs (Zhu *et al*, 2005). For uterine corpus cancer, we adjusted for the joint effects of parity and cigarette smoking with age-specific RRs (Brinton *et al*, 1992; Viswanathan *et al*, 2005). As a result of sparse data, we separately evaluated the effect on uterine corpus cancer of BMI (Ballard-Barbash and Swanson, 1996). We did not adjust for effects of oral contraceptive use because prevalence of use did not vary materially between the AIDS patients and the NHIS (data not presented).

## RESULTS

In linkages performed during 2003–2005 in 12 regions of the US with population-based cancer ascertainment, 85 268 women were followed for 665 987 person-years, measured from 5 years before to 10 years after an initial AIDS diagnosis. There were 51 616 women of African ancestry (61%), 17 705 women of Hispanic ancestry (21%), 15 019 non-Hispanic women of European ancestry (18%), and 928 women of other or missing ancestry (1%). At AIDS diagnosis, median age of the women was 36 years (interquartile range 31–43 years); 90% of the women were age 50 years or younger.

The incidence of breast and uterine corpus cancers, but not of ovary cancer, was significantly lower than in the general population. Specifically, linkage to the cancer registries revealed 313 cases of breast cancer (SIR 0.69; 95% CI, 0.62–0.77), 31 cases of uterine corpus cancer (SIR 0.57; 95% CI, 0.39–0.81), and 42 cases of ovary cancer (SIR 1.05; 95% CI, 0.75–1.42) among women with AIDS. Breast cancer SIR was reduced in all groups defined by age at AIDS onset, including those with AIDS onset before age 35 (SIR 0.69). As shown in Table 1, the SIR for breast and ovary cancers did not differ by age at cancer diagnosis, a surrogate for menopause status. In contrast, the uterine corpus cancer SIR differed by menopause status: 0.86 (95% CI, 0.54–1.32) before age 50 and 0.33 (95% CI, 0.16–0.61) at or after age 50. Standardized incidence ratio did not vary by racial ancestry for any of the three cancers (Table 1). Cancer and AIDS records matched exactly on social security number for 211 (67%) of the breast cancer cases; the records for the other 102 cases matched on other criteria. Breast cancer SIR did not vary by availability of social security number for matching (Table 2). By stage, breast cancer SIR from 4 to 60 months after AIDS onset was 0.49 (95% CI, 0.34–0.68) for locally invasive cancer, 0.61 (95% CI, 0.42–0.86) for regional dissemination, and 0.89 (95% CI, 0.40–1.68) for distant metastases (Table 2). Data for uterine corpus and ovary cancer were too sparse to evaluate by stage.

We examined trends in SIR with respect to time relative to AIDS onset and CD4 count, as measures of immune deficiency (Table 2). Near the time of the initial AIDS-defining condition (−6 to +3 months), the SIR was 1.15 (95% CI, 0.87–1.50) for breast cancer, 1.19 (95% CI, 0.48–2.45) for uterine corpus cancer, and 2.90 (95% CI, 1.54–4.94) for ovary cancer. Excluding this interval of intensive diagnostic scrutiny, there was no significant trend in the breast cancer SIR from 60 months before to 120 months after AIDS onset (*P*_trend_=0.14, Table 2). There was no trend with AIDS-relative time in the SIR for uterine corpus cancer or ovary cancer (*P*_trend_=0.72 and 0.94, respectively, data not presented). As with the AIDS-relative time analyses, CD4 lymphocyte count at AIDS onset was not significantly associated with SIR for breast cancer (*P*_trend_=0.47, Table 2) or, based on sparse data, for uterine corpus or ovary cancer (*P*_trend_=0.91 and 0.89, respectively, data not presented).

**Figure 1:**
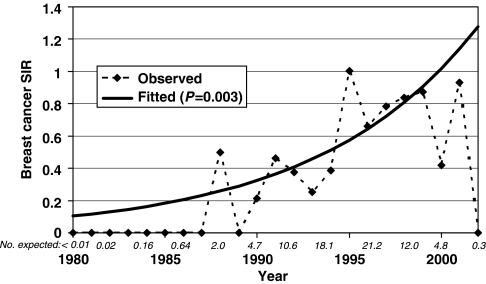
Observed and fitted (linear Poisson regression model) standardized incidence ratio (SIR) for breast cancer occurring 1–5 years after AIDS diagnosis (*n*=90 observed), by calendar year. A quadratic Poisson regression model did not fit the data better (*P*=0.10) than this linear model. The number of cancer cases expected in each even-number year is shown in italics.

Calendar time was used as a surrogate for availability of anti-HIV therapy – little or none pre-1990, single and dual reverse transcriptase inhibitors in 1990–1995, and HAART after 1995. As shown in Table 2, for breast cancer occurring 4 to 27 months after AIDS onset, no cases occurred (*vs* 4.7 expected) in the cohort with AIDS onset in 1980–1989, whereas the SIR was 0.38 with AIDS onset in 1990–1995, and the SIR was 0.83 with AIDS onset in 1996–2002 (*P*_trend_=0.002). Excluding the 1980–1989 cohort that had no cases, the SIR still increased significantly (*P*=0.01) from the 1990–1995 to the 1996–2002 cohort. The significant increase in breast cancer SIR also was found using single calendar years in a Poisson regression model (*P*_trend_=0.003, Figure 1). Data were very sparse for years 2000–2002, with only 7.2 breast cancer cases expected. Comparing the 1990–1995 and the 1996–2002 cohorts, risk of local stage breast cancer increased from SIR 0.40 (95% CI, 0.22–0.66) to 0.61 (95% CI, 0.38–0.93). Similarly, risk of regional stage breast cancer risk increased from SIR 0.53 (95% CI, 0.29–0.88) to 0.77 (95% CI, 0.47–1.19). There was no trend with calendar time in the SIR for uterine corpus cancer, ovary cancer, or distant metastatic breast cancer (*P*_trend_⩾0.5) but data were sparse.

Indirect adjustment for age at first live birth, nulliparity and BMI had little effect on the breast cancer SIR estimate (SIR_adj_ 0.72 *vs* 0.69 unadjusted). For uterine corpus cancer overall, there was little effect with indirect adjustment for parity and smoking (SIR_adj_ 0.59) or for BMI (SIR_adj_ 0.56) compared to the unadjusted estimate (SIR 0.57). Among postmenopausal women, the SIR for uterine corpus cancer was modestly attenuated with adjustment for parity and smoking (SIR_adj_ 0.39) and for BMI (SIR_adj_ 0.37, compared to the unadjusted SIR 0.33).

## DISCUSSION

Women with AIDS had a low incidence of locally invasive and regionally disseminated breast cancer, but this cancer deficit attenuated over time to approach the incidence in the general population. Ovary cancer incidence did not differ with AIDS, but a uterine corpus cancer deficit was large and statistically significant among postmenopausal women. Although women with HIV/AIDS had lower fertility and BMI in the pre-HAART era (Lee *et al*, 2000; Forsyth *et al*, 2002), the breast cancer deficit with AIDS was minimally altered by adjustment for these factors, and it was unrelated to CD4 count or AIDS-relative time. A breast cancer deficit with AIDS has been noted inconsistently in previous, much smaller studies (Franceschi *et al*, 1998; Amir *et al*, 2000; Grulich *et al*, 2002; Herida *et al*, 2003; Hessol *et al*, 2004).

Cancer risk with HIV/AIDS might reflect differences in hormonal balance or cycling. In the general population, high levels of serum estrogens and testosterone have been linked with an increased risk of breast cancer in postmenopausal women (Key *et al*, 2002). Although serum levels of sex hormones have not been convincingly linked to premenopausal breast cancer (Sturgeon *et al*, 2004), lower breast cancer risk with increased physical activity, participation in college athletics, or anorexia nervosa, which result in infrequent ovulation or shortened luteal phase cycles (Bernstein *et al*, 1994; Wyshak and Frisch, 2000; Michels and Ekbom, 2004), suggests that premenopausal sex hormones also contribute to breast cancer risk. In women with HIV-related CD4 lymphocyte counts of 200–500 cells *μ*l^−1^, total estradiol and testosterone levels were at the low end of the normal range (Miller *et al*, 1998). Of note, free testosterone levels were below normal in one-third of HIV-infected women with normal weight, half of those with up to 10% of body weight loss, and two-thirds of those with more severe weight loss, of whom 38% were amenorrheic (Grinspoon *et al*, 1997). Generally, however, menstrual cycles of HIV-infected women are normal or only slightly irregular (Ellerbrock *et al*, 1996).

Since the 1980s, HIV/AIDS patients have had marked changes in body composition that may have affected their endogenous hormone levels and risk of cancer. In the early 1990s, before availability of HAART and the current obesity epidemic (Flegal *et al*, 2002), one-fifth of HIV-infected women in New York City reported losing more than 10% of their usual body weight (Ellerbrock *et al*, 1996). Then, during the first year on HAART, BMI increased dramatically, almost entirely with fat (Silva *et al*, 1998). During the HAART era, HIV-infected women in the US have tended to be overweight or obese (Shuter *et al*, 2001; Mulligan *et al*, 2005). A single BMI value, as we used for adjustment, would not reflect these substantial and rapid changes. Thus, attenuation of the breast cancer deficit may still be tied, in part, to increases in body fat as anti-HIV treatments have been increasingly effective, available, and used (Herida *et al*, 2003).

Perhaps the deficit in breast cancer reflects the ability of HIV to infect, replicate in, and impair proliferation of breast cells (Toniolo *et al*, 1995; Southern, 1998). Epithelial cell infection by HIV is inefficient but occurs through several CD4-independent mechanisms (Phillips and Bourinbaiar, 1992). Likewise, HIV can transiently infect epithelial and stromal cells of the endometrium, with notable differences in HIV receptor expression and cytotoxic T-lymphocyte activity by menstrual phase and menopause status (Howell *et al*, 1997; Asin *et al*, 2003; Yeaman *et al*, 2003). Anti-HIV medications would likely reduce HIV replication and perhaps allow cell proliferation, either directly or indirectly (Asin *et al*, 2003; Bettaccini *et al*, 2005; Chung *et al*, 2005). If so, increasing use and effectiveness of anti-HIV treatments would attenuate the cancer deficit.

Bias is an important concern for our study. Women with HIV/AIDS may have been screened infrequently and had conditions that diverted attention from breast masses or vaginal bleeding (Preston-Martin *et al*, 2002). The smaller deficit with distant metastases (SIR 0.89) than earlier stage breast cancer (SIR 0.54) implies delayed diagnosis. Screening bias would not readily account for a breast cancer deficit among young women with AIDS (SIR 0.69), as routine mammograms are not recommended before age 40. Screening also is not performed for uterine corpus cancer. Cancer deficits could be an artefact of cases missed in the registry match. As many women change names upon marriage or divorce, sensitivity of the linkage might be lower than the 95% reported previously (Borges *et al*, 2001). However, many records were linked with social security number, and the breast cancer deficit was unrelated to completeness of records with social security numbers available for matching. Finally, based on undetected deaths with central nervous system lymphoma (unpublished data), we estimate that 15% of the deficit may reflect migration out of the registration area (Cote *et al*, 1997).

From 1980 to 2002, women with AIDS had significantly reduced risk of breast and postmenopausal uterine corpus cancers, but not of ovary cancer. The cancer deficits were unrelated to immune deficiency or selected risk factors. We offer two hypotheses for the breast cancer deficit. First, women with HIV/AIDS had alterations of reproductive and perhaps other hormones tied to breast cancer (Rose *et al*, 2004). Second, HIV infected the breast, impairing proliferation of malignantly transformed cells. Both mechanisms would be reduced with effective anti-HIV therapy, resulting in an increased incidence of breast cancer that approaches that of the general population.

## Figures and Tables

**Table 1:** Standardized incidence ratio (SIR) of cancer among 85 268 women from 60 months before to 120 months after an initial AIDS-defining event, 1980–2002^a^

	**Cancer cases**		**SIR**
**Cancer type subgroup^b^**	**Observed**	**Expected**	**(95% CI)**
Breast cancer	313	453.3	0.69 (0.62–0.77)
Premenopausal	201	283.6	0.71 (0.61–0.81)
Postmenopausal	112	169.7	0.66 (0.54–0.79)
African ancestry	181	248.1	0.73 (0.63–0.84)
Other ancestry	132	205.2	0.64 (0.54–0.76)
			
Uterine corpus cancer	31	54.6	0.57 (0.39–0.81)
Premenopausal	21	24.3	0.86 (0.54–1.32)
Postmenopausal	10	30.3	0.33 (0.16–0.61)
African ancestry	13	25.0	0.52 (0.28–0.89)
Other ancestry	18	29.6	0.61 (0.36–0.96)
			
Ovary cancer	42	40.1	1.05 (0.75–1.42)
Premenopausal	28	25.6	1.09 (0.73–1.58)
Postmenopausal	14	14.5	0.97 (0.53–1.62)
African ancestry	23	18.5	1.25 (0.79–1.87)
Other ancestry	19	21.7	0.88 (0.53–1.37)

a The 85 268 women, aged 15–91, accumulated 665 987 person-years, censored at death and excluding time before and after the population-based cancer registry in the same region had complete data.

b Other ancestry includes European (including all Hispanics), other, and missing ancestry. Premenopausal are cancers occurring before age 50. Postmenopausal are cancers occurring at or after age 50.

**Table 2:** Standardized incidence ratio (SIR) of breast cancer among women with AIDS

	**Breast cancer cases**			
	**Observed**	**Expected**	**SIR**	**95% CI**
*Matches by social security number* a				
High (84%)	111	150.4	0.74	0.61–0.89
Medium (70%)	149	225.6	0.66	0.56–0.78
Low (25%)	53	77.1	0.69	0.51–0.90
				
*Cancer stage* b				
Local	37	75.6	0.49	0.34–0.68
Regional	34	55.4	0.61	0.42–0.86
Distant	9	10.2	0.89	0.40–1.68
Missing/unknown	7	8.7	0.81	0.32–1.66
				
*AIDS-relative time (months)* c				
−60 to –25	64	119.9	0.53	0.41–0.68
−24 to −7	83	84.1	0.99	0.79–1.22
−6 to +3	56	48.5	1.15^d^	0.87–1.50
+4 to +27	47	81.1	0.58	0.43–0.77
+28 to +60	40	70.5	0.57	0.41–0.77
+61 to +120	23	49.2	0.47	0.30–0.70
	*P*_trend_ 0.14			
				
*CD4 count* e				
0–99 cells *μ*l^−1^	24	44.7	0.54	0.34–0.80
100–199 cells *μ*l^−1^	31	51.5	0.60	0.41–0.85
⩾200 cells *μ*l^−1^	12	17.4	0.69	0.35–1.21
	*P*_trend_ 0.47			
				
AIDS onset year^f^				
1980–1989	0	4.7	0	0–0.78
1990–1995	14	36.7	0.38	0.21–0.64
1996–2002	33	39.6	0.83	0.57–1.17
	*P*_trend_ 0.002			

a Four registries in ‘high’, three in ‘medium, five in ‘low’. Excludes rural Georgia, which had no matched breast cancer cases.

b Cancer risk by stage during +4 to +60 months after AIDS onset. One registry with miscoded stage data excluded.

c AIDS-relative time intervals are: distant pre-AIDS (−60 to −25), later pre-AIDS (−24 to −7), at AIDS onset (−6 to +3, excluded from trend test), shortly after AIDS onset (+4 to +27), later after AIDS onset (+28 to +60), and very late after AIDS onset (+61 to +120).

d *P*_trend_ for AIDS-relative time excludes the AIDS onset period (−6 to +3 months).

e Observed and expected cases and SIR presented are for +4 to +60 months by CD4 count at AIDS (within −6 to +3 months of AIDS onset). Results for breast cancer were similar during +4 to +27 months (*P*_trend_=0.63) and +28 to +60 months (*P*_trend_=0.58).

f Observed and expected cases and SIR presented are for +4 to +27 months by cohort of AIDS onset: little or no antiretroviral therapy (1980–1989), availability of single and dual nucleoside reverse transcriptase inhibitors (1990–1995), and availability of highly active antiretroviral therapy (HAART) combinations (1996–2002). Breast cancer trend by calendar year of AIDS onset was similar in two sensitivity analyses. For one, cases occurring in the +4 to +60 post-AIDS interval were used (*P*_trend_=0.01). For the second, using the +4 to +27 post-AIDS interval, AIDS-onset years were divided as 1980–1986, 1987–1995, and 1996–2002 (*P*_trend_=0.006).
